# Endostatin, an angiogenesis inhibitor, ameliorates bleomycin-induced pulmonary fibrosis in rats

**DOI:** 10.1186/1465-9921-14-56

**Published:** 2013-05-20

**Authors:** Yun-Yan Wan, Guang-Yan Tian, Hai-Sheng Guo, Yan-Meng Kang, Zhou-Hong Yao, Xi-Li Li, Qing-Hua Liu, Dian-Jie Lin

**Affiliations:** 1 Department of Respiratory Medicine, Shandong Provincial Hospital, Shandong University, Jinan, Shandong Province 250021, People's Republic of China; 2 Department of Neurology, Jinan Children's Hospital, Jinan, Shandong Province 250021, People's Republic of China; 3 Department of Respiratory Medicine, Dongying People's Hospital, Dongying, Shandong Province 257000, People's Republic of China

**Keywords:** Endostatin, Bleomycin, Pulmonary fibrosis, Angiogenesis, Vascular endothelial growth factor, Extracellular signal-regulated protein kinase 1/2, Inflammation, Epithelial cell apoptosis

## Abstract

**Background:**

Recent evidence has demonstrated the role of angiogenesis in the pathogenesis of pulmonary fibrosis. Endostatin, a proteolytic fragment of collagen XVIII, is a potent inhibitor of angiogenesis. The aim of our study was to assess whether endostatin has beneficial effects on bleomycin (BLM)-induced pulmonary fibrosis in rats.

**Methods:**

The rats were randomly divided into five experimental groups: (A) saline only, (B) BLM only, (C) BLM plus early endostatin treatment, (D) BLM plus late endostatin treatment, and (F) BLM plus whole-course endostatin treatment. We investigated the microvascular density (MVD), inflammatory response and alveolar epithelial cell apoptosis in rat lungs in each group at different phases of disease development.

**Results:**

Early endostatin administration attenuated fibrotic changes in BLM-induced pulmonary fibrosis in rats. Endostatin treatment decreased MVD by inhibiting the expression of VEGF/VEGFR-2 (Flk-1) and the activation of extracellular signal-regulated protein kinase 1/2 (ERK1/2). Endostatin treatment also decreased the number of inflammatory cells infiltrating the bronchoalveolar lavage fluid during the early inflammatory phase of BLM-induced pulmonary fibrosis. In addition, the levels of tumour necrosis factor-α (TNF-α) and transforming growth factor β1 (TGF-β1) were reduced by endostatin treatment. Furthermore, endostatin decreased alveolar type II cell apoptosis and had an epithelium-protective effect. These might be the mechanism underlying the preventive effect of endostatin on pulmonary fibrosis.

**Conclusions:**

Our findings suggest that endostatin treatment inhibits the increased MVD, inflammation and alveolar epithelial cell apoptosis, consequently ameliorating BLM-induced pulmonary fibrosis in rats.

## Background

Idiopathic pulmonary fibrosis (IPF) is a chronic and disabling lung disease with a high mortality rate due to ultimate respiratory failure [1]. The mean survival time varies from 3.2 to 5 years after diagnosis [2,3]. The therapeutic options are limited and often disappointing [4-6]. Although the aetiology remains unknown, aberrant angiogenesis plays an important role in the development of pulmonary fibrosis. Many studies have demonstrated that the inhibition of vascular remodelling attenuates pulmonary fibrosis in animal models [7-13].

Among the numerous cytokines related to angiogenesis, vascular endothelial growth factor (VEGF) has been recognised as an important regulator of angiogenesis and a major enhancer of vascular permeability in several types of inflammatory lesions [10,14]. It is essential for endothelial cell survival, proliferation and tube formation. The biological functions of VEGF polypeptides result from binding to two cellular receptors, VEGFR1 (Flt-1) and VEGFR2/KDR (Flk-1), on endothelial cells. Signalling through Flk-1 is responsible for the vascular homeostasis and survival functions of VEGF [14]. Furthermore, VEGF has been reported to recruit leukocytes to sites of inflammation, neovascularisation, and vascular injury by stimulating the expression of MCP-1 [15]. VEGF regulates endothelial cell morphology and activates multiple signal transduction pathways, including extracellular signal–regulated kinase (ERK1/2) [16], which is involved in regulating the angiogenesis and inflammation response in the lung. A previous study [17] confirmed that VEGF exacerbates pulmonary fibrosis, suggesting that VEGF facilitates the fibrogenic process. VEGF inhibition is being tested as a strategy for the prevention of angiogenesis and vascular leakage in pulmonary fibrosis.

Endostatin, a 20 kDa carboxyl-terminal proteolytic fragment of collagen XVIII, has been thoroughly studied as a potent inhibitor of angiogenesis since its discovery by O’Reilly et al. in 1997 [18]. Endostatin inhibits endothelial cell proliferation, migration/invasion, and tube formation [19] and induces endothelial cell apoptosis [20]. Endostatin has been shown to inhibit new blood vessel formation under different pathological conditions characterised by increased angiogenesis, such as tumours [18], endometriosis [21], arthritis [22], ulcerative colitis [23] and other diseases in many experimental models.

Endostatin interferes with VEGF/VEGFR signalling [24,25]. Endostatin has been reported to ameliorate peritoneal sclerosis by reducing expression of transforming growth factor β1 (TGF-β1) [26], which is the most important profibrotic growth factor.

In addition, endostatin suppresses the production of the proinflammatory cytokine tumour necrosis factor-α (TNF-α) [22]. VEGF, TGF-β1 and TNF-α are overexpressed in the early stages of pulmonary fibrosis. Isobe [27] found that endostatin neutralisation treatment in a rat myocardial infarction (MI) model resulted in increased angiogenesis, exaggerated tissue remodelling and interstitial fibrosis in post-MI hearts. On the basis of these reports, we hypothesise that endostatin may have protective effects, particularly by inhibiting angiogenesis, in the pathogenesis of pulmonary fibrosis. To test this hypothesis, the in vivo effect of endostatin on BLM-induced lung injury and fibrosis in rats was investigated. We assessed the antiangiogenic efficacy of endostatin treatment and also investigated its effect on VEGF signal transduction. We show that endostatin prevents BLM-induced pulmonary fibrosis in rats through the reduction of aberrant angiogenesis, proinflammatory cytokine production and alveolar epithelial cell apoptosis.

## Methods

### Animals and drugs

One hundred pathogen-free 8-week-old Sprague–Dawley (SD) male rats weighing 200–250 g were purchased from the Laboratory Animal Center, Shandong University of traditional Chinese medicine. This study was approved by the Shandong Animal Care and Use Committee and followed the national and institutional rules concerning animal experiments. Rats were housed at 23 ± 2°C and 55 ± 5% humidity, with a 12 h light–dark cycle. Rodent food and water were provided ad libitum. The rats were maintained for 1 week before the start of experiments to adapt to the environment. Experimental animals were treated in accordance with the criteria outlined in the Guide for the Care and Use of Laboratory Animals.

Bleomycin hydrochloride, purchased from Tianjin Taihe Pharmaceutical Co. (Tianjin, China), was dissolved in sterile 0.9% saline on the day of intratracheal instillation at a dose of 5 mg/kg per rat. Recombinant human endostatin was provided by Jiangsu Simcere Medgenn Bio-Pharmaceutical Co. Ltd. (Nanjing, China), and injected at a dose of 2 mg/kg body weight per rat.

### Animal model of BLM-induced pulmonary fibrosis

The rats were randomly divided into five groups: (A) saline only (control group, SA group); (B) BLM only (BLM group); (C) BLM plus early endostatin treatment (E-ES group); (D) BLM plus late endostatin treatment (L-ES group); and (F) BLM plus whole-course endostatin treatment (W-ES group).

Animals were anaesthetised with an intraperitoneal injection of sodium pentobarbital (20 mg/kg) and then given an intratracheal instillation of 5 mg/kg BLM. The rats were rotated immediately after BLM instillation to ensure thorough drug distribution in the lungs. Control animals received an equal volume of sterilised saline using the same procedure. After recovery from the anaesthesia, the rats were returned to their cages and allowed food and water as normal.

To clarify the effect of endostatin on different stages of BLM-induced pulmonary fibrosis development, endostatin was administered daily through subcutaneous injection (2 mg/kg body weight) at the early phase (from days 1–14), the late phase (from days 15–28) or during the entire course (from days 1–28) after BLM infusion. Rats from each group were divided into four subgroups, which were sacrificed on days 3, 7, 14 and 28. Blood was collected from the abdominal aorta and centrifuged at 2500 rpm for 10 min. The serum was stored at −80°C.

### Histological examination

After sacrifice, the left lungs were fixed in 4% paraformaldehyde, dehydrated, and embedded in paraffin. The tissues were then sectioned at 5 μm and stained with haematoxylin & eosin (H&E) and Masson's trichrome to examine the degree of fibrosis. Ten fields in each section were analysed. The severity of fibrotic changes in each histological section of the lung was assessed as the mean score of severity from the observed microscopic fields using the semiquantitative grading system described by Ashcroft and co-workers [28].

### Hydroxyproline assay of lung tissues

Hydroxyproline was measured using the hydroxyproline test kit from the Nanjing Jiancheng Bioengineering Institute according to the manufacturer’s instructions. In brief, 30–100 mg of lung tissue was hydrolysed in 1 ml lysis buffer solution (pH 7.4, 10 mM Tris–HCl, 0.1 mM EDTA–2Na, 10 mM saccharose, and 0.8% sodium chloride solution) at 100°C for 20 min, and the hydroxyproline concentration was determined according to Otsuka’s previously published method [29]. Data were expressed as micrograms of hydroxyproline per gram of wet lung weight.

### Immunohistochemical analysis

After deparaffinisation in xylene and hydration with graded alcohol, the sections were incubated in citrate buffer (pH 6.0) at 96°C for 20 min to retrieve the antigen. After cooling at room temperature, the sections were placed in 3% hydrogen peroxide (H_2_O_2_) for 15 min at room temperature to inactivate endogenous peroxidases. After washing with PBS three times, the sections were blocked with rabbit serum for 30 min, followed by incubation with primary antibodies against the CD31 antigen (1:200, Santa Cruz Biotechnology, California, USA) and Flk-1 (1:200, Beijing Biosynthesis Biotechnology Co., Ltd., China) at 4°C overnight. After washing with PBS, the sections were incubated in biotinylated rabbit anti-goat antibody for 30 min at 37°C. Then, the sections were washed in PBS, and the ABC reagent was applied (Wuhan Boster Biological Technology Ltd, China). Antibody binding was detected with a diaminobenzidine kit, and the sections were counterstained with haematoxylin.

Microvessel density (MVD) was assessed by two independent observers as reported by Weidner [30]. Briefly, MVD was recorded as the number of CD31-positive endothelial cells or endothelial cell clusters per high-power field (× 200) from the five areas of highest vascularisation. Vessels with thick muscular walls were excluded. Flk-1 was evaluated using immunostaining scores (range 0–5), which were calculated by adding the distribution (none = 0, focal = 1, multifocal = 2, and diffuse = 3) to the intensity of the staining (none = 0, mild = 1, and strong = 2) [31].

### Bronchoalveolar lavage

After the rats were sacrificed by arterial exsanguination, the chest cavity was opened with a midline incision. The right main bronchus was ligated. Bronchoalveolar lavage was performed only from the left lung through a tracheal cannula with 3 ml cold PBS (pH 7.4). This procedure was repeated five times. Fluid recovery was consistently above 85%. Total cell counts were obtained in bronchoalveolar lavage fluid (BALF) using a haemocytometer. After centrifugation at 4°C and 1000 × g for 10 min, the cell-free supernatant was collected and stored at −80°C for biochemical measurements. The cell pellet was resuspended in cold PBS, smeared on a glass slide, air dried, and stained with Wright-Giemsa to identify different cell types. Two hundred cells from each animal were counted and expressed as a percentage of total cells. The protein permeability index (PPI) was calculated as BALF total protein/plasma total protein × 100, as described previously [32].

### Real-time PCR

Total RNA was extracted from frozen lung tissues using Trizol (Takara Bio Inc. Japan) according to the manufacturer's instructions. Real-time PCR was performed with a Lightcycler480 (Roche) using the SYBR Green assay after reverse transcribing 1 μg of RNA with reverse transcriptase. All the results were normalised to the levels of β-actin mRNA. The primer pairs and expected lengths (in bp) were as follows (5' to 3'): VEGFA (GenBank: NM_001110333.1, forward: GTCCTCACTTGGATCCCGACA, reverse: CCTGGCAGGCAAACAGACTTC; 99 bp), Flk-1 (GenBank: NM_013062.1, forward: AATGCCCATGACCAAGAATGTG, reverse: GGATAGAGCCGCGTGTCTGAA; 129 bp), and β-actin (GenBank: NM_031144.3, forward: CTAAGGCCAACCGTGAAAAGA, reverse: CCAGAGGCATACAGGGACAAC; 103 bp). The cycling conditions were as follows: 95°C for 30 s, followed by 40 cycles of 95°C for 5 s, 65°C for 30 s, and 65°C for 15 s. Relative gene expression was calculated as 2^−ΔΔCT^[33].

### Enzyme-linked immunosorbent assay analysis

The concentrations TGF-β1 (active form) and VEGF in lung tissue lysates and TNF-α in BALF were measured. Briefly, 100 mg of lung tissue from both control and treated animals was homogenised in 1 ml 50 mM Tris–HCl (pH 7.4) containing 1% NP-40, 50 mM NaCl, 0.5 mM ethylene diamine tetraacetic acid (EDTA), and 100 mM phenylmethylsulfonyl fluoride (PMSF) [34]. The protein concentration in each sample was determined using the bicinchoninic acid (BCA) method according to the manufacturer’s instructions. Levels of TGF-β1, VEGF and TNF-α were measured using quantitative ELISA kits according to the manufacturer’s protocols (R&D Systems, Minneapolis, MN).

### Western blot

Protein samples (40 μg) were separated on precast 10% SDS polyacrylamide gels (SDS-PAGE). After electrophoresis, the proteins were transferred to PVDF membrane filters (Millipore Biotechnology Inc., USA). The membranes were incubated overnight at 4°C with primary rabbit polyclonal phosphorylated ERK1/2 (pERK1/2) and ERK (1:2000 dilutions in 5% BSA in TBS-T; Cell Signaling, Beverly, MA). After washing three times in TBS-T, horseradish peroxidase (HRP)-conjugated secondary antibodies were used at a dilution of 1:5000 in TBS-T for 1 h at room temperature. After three additional washes with TBS-T, the immunoreactive bands were visualised with a chemiluminescence reagent (ECL, Millipore Biotechnology Inc., USA) and quantified using Multi Gauge V3.2 (Fuji Film, Japan) Analysis Software.

### Quantitation of alveolar epithelial cell apoptosis

To evaluate the apoptosis of alveolar epithelial cells, terminal deoxynucleotidyl transferase-mediated dUTP nick end labeling (TUNEL) and immunofluorescent double-staining of surfactant protein C (SP-C) were performed on lung sections on days 7 and 28. TUNEL assay was conducted by using the In Situ Cell Death detection kit (TMR red, Roche, Mannheim, Germany) according to the manufacturer’s instructions. Briefly, deparaffinized sections were permeablized with proteinase K (20 *μ*g/ml in PBS) for 15 min at 37°C. After washing with PBS, the sections were then treated with TUNEL reaction mixture containing TdT and fluorescein isothiocyanate-labelled dUTP for 60 min at 37°C. SP-C (Wuhan Boster Biological Technology Ltd, China) was used to identify alveolar epithelial cells. Goat anti-rabbit IgG-FITC was used as the secondary antibody. The nuclei were stained with 4', 6'-diamidino-2-phenylindole (DAPI). Ten high-power fields of each section were randomly selected under a fluorescence microscope, and the numbers of TUNEL-positive cells and SP-C-positive cells were recorded [35].

### Statistical analysis

All results were expressed as the mean ± SD. A two-way ANOVA with multiple comparison tests to assess differences between groups was performed. All data analysis was performed using SPSS 18.0 software. Statistical graphs were generated with GraphPad Prism 5 software. P values less than 0.05 were considered statistically significant.

## Results

### Effect of endostatin on BLM-induced pulmonary fibrosis

**Figure 1:**
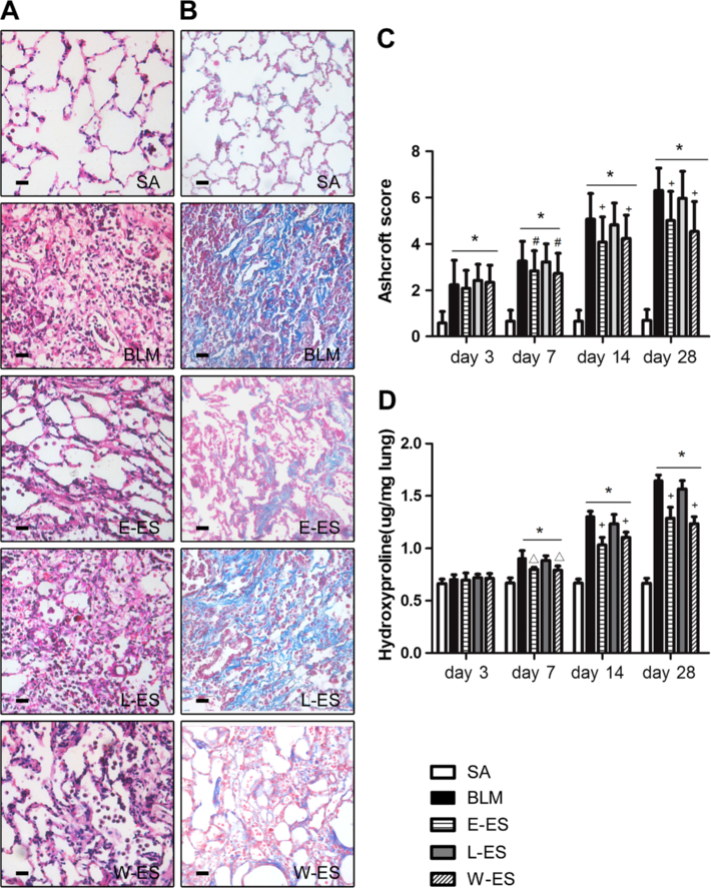
**Effect of endostatin on BLM-induced pulmonary fibrosis.** (**A**) Representative images of hematoxylin and eosin (H&E) and (**B**) Masson trichrome –stained sections of rats in each experimental group on day 28 (×200). (**C**) Comparison of the Ashcroft score among the experimental groups. (**D**) Collagen deposition was assessed by measuring the hydroxyproline content. Bar = 100μm. Results are expressed as mean ± SD, n = 5 in each group, *P < 0.001 vs SA group; #P < 0.05 vs BLM group; △P < 0.01 vs BLM group; +P < 0.001 vs BLM group.

We evaluated the effect of endostatin treatment in the BLM-induced lung fibrosis model. Lung fibrosis was assessed by morphometry of Masson's trichrome-stained lung sections and hydroxyproline deposition in lung tissue. Masson and H&E staining demonstrated that BLM treatment significantly induced distortion of the lung structure and collagen deposition in the lungs, whereas well-alveolised normal histology was observed in the saline control group (Figure 1A,B). The administration of endostatin for the first 14 days or throughout the course of the experiment effectively ameliorated BLM-induced pulmonary fibrosis compared to the control, whereas the histopathological characteristics of the L-ES group were not significantly different from the BLM group. To grade the extent of lung fibrosis, the fibrotic changes in the lung were assessed using the Ashcroft score. The scores of the E-ES and W-ES groups were significantly lower than the BLM group on days 7, 14 and 28 after BLM administration (Figure 1C).

The collagen content of rat lungs was assessed using the hydroxyproline assay. The concentration of hydroxyproline in the lung tissue at 28 days after BLM injection was significantly increased in the BLM group compared with the saline control group (Figure 1D). In the E-ES and W-ES groups, the hydroxyproline content was significantly decreased compared with the BLM group on days 7, 14 and 28. No significant difference in Ashcroft score or hydroxyproline content was observed in the L-ES group compared to the BLM group. The hydroxyproline content was lower in the L-ES group than in the BLM group, although this difference was not statistically significant (P = 0.09 on day 28).

### Effect of endostatin on the inflammation response in BLM-induced pulmonary fibrosis

The early response to BLM challenge is characterised by a dramatic increase in inflammatory cell recruitment. To investigate the effect of endostatin on the inflammation response induced by BLM infusion in rats, we assessed the inflammatory cell count and the concentration of the pro-inflammatory cytokine TNF-α in BALF.

**Figure 2:**
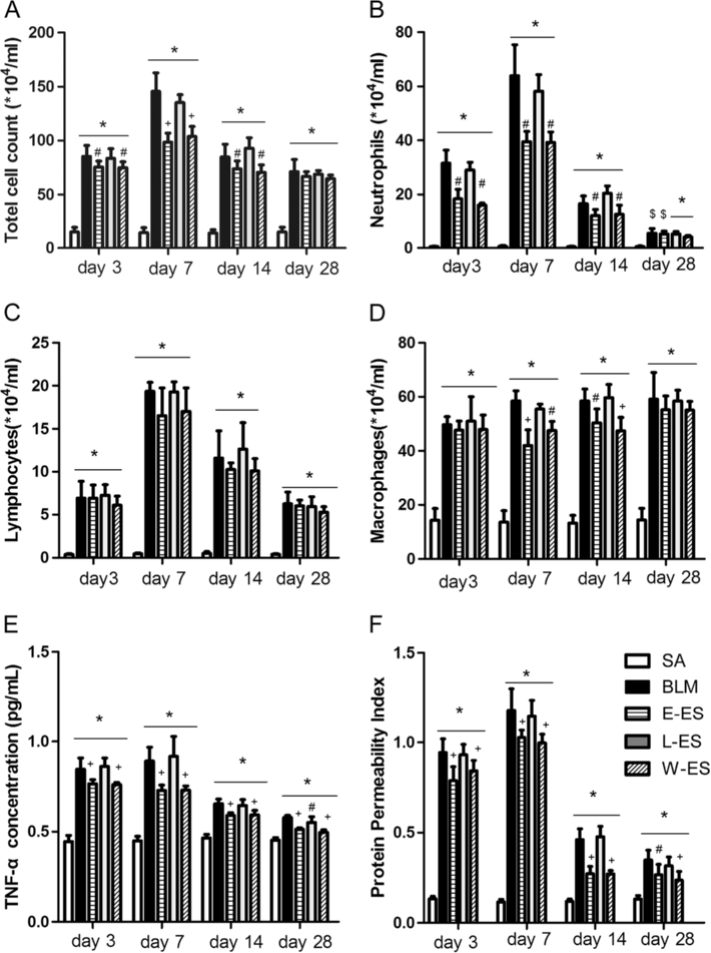
**Effect of endostatin on the inflammatory response in BLM-induced pulmonary fibrosis.** Changes in (**A**) total cell count, (**B**) neutrophils, (**C**) lymphocytes, (**D**) macrophages and (**E**) TNF-α expression in BALF of rats at each stage were presented. (**F**) Representative PPI change, calculated as BALF total protein/plasma total protein × 100. Results are expressed as mean ± SD, n = 4 or 5 in each group, *P < 0.001 vs SA group; $P < 0.05 vs SA group; #P < 0.05 vs BLM group; +P < 0.001 vs BLM group.

The total and differential cell counts in BALF for each day were presented in Figure 2. The total number of cells was significantly increased at all time points in the BLM-treated rats. The number of neutrophils in the BALF after BLM infusion peaked on day 7 and decreased from day 14 to day 28 but was still elevated compared to the saline control group. Treatment with endostatin significantly inhibited the accumulation of total leukocytes in BALF on days 3, 7 and 14 after BLM challenge (Figure 2A), which appeared to be caused by a significant decrease in neutrophil recruitment on days 3, 7 and 14 (Figure 2B). There was no change in lymphocyte number between BLM groups and the groups administered endostatin (Figure 2C). The number of macrophages was significantly decreased by endostatin treatment on days 7 and 14 (Figure 2D). No differences in total or differential cell count were observed between the L-ES and BLM groups.

The peak concentration of TNF-α in BALF in the BLM group was observed on day 3, and the concentration gradually declined on days 7, 14 and 28, although levels remained significantly higher than in the saline group at each timepoint studied. Compared with the BLM group, the concentrations of TNF-α in the E-ES and W-ES groups were significantly decreased (Figure 2E), and late endostatin administration produced a similar effect (P < 0.05 on day 28).

Vascular permeability plays a key role in leukocyte and protein leakage. In our study, we assessed the PPI as the ratio of BALF to plasma protein, which was also suppressed by endostatin administration at early stages in the E-ES and W-ES groups, as depicted in Figure 2F.

### Effect of endostatin on MVD in BLM-treated rat lung tissues

**Figure 3:**
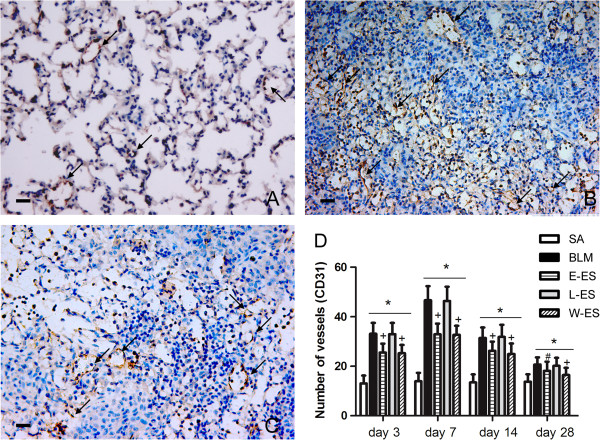
**Effect of endostatin on MVD in BLM-induced pulmonary fibrosis.** Immunohistochemical staining of CD31 in lung sections in SA (**A**), BLM (**B**) and E-ES (**C**) groups on day 7 (×200). (**D**) MVD was assayed by counting the number of the microvessels per high power field in lung sections stained with antibody CD31. Bar = 100μm. *P < 0.001 vs SA group; #P < 0.01 vs BLM group; +P < 0.001 vs BLM group.

Endostatin possesses antiangiogenic activities. To explore the effect of endostatin on angiogenesis in BLM-induced pulmonary fibrosis, we quantified MVD in the lung by immunostaining for the endothelial marker CD31. The number of CD31-positive vessels was increased in BLM-treated lungs on day 3 and peaked at day 7, gradually declining with the aggravation of lung fibrosis. Endostatin administration significantly decreased the number of CD31-positive cells at each stage compared to the BLM-only group levels (Figure 3). The antiangiogenic effect was particularly striking on day 7.

### Effect of endostatin on VEGF/Flk-1 expression in BLM-induced pulmonary fibrosis

**Figure 4:**
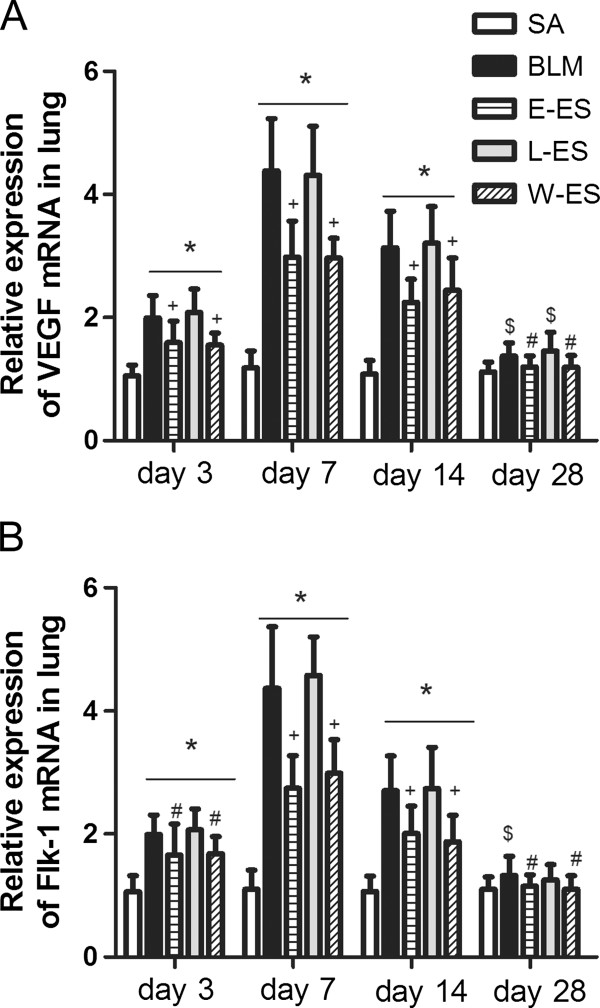
**Effect of endostatin on VEGF/Flk-1 mRNA expression in BLM-induced pulmonary fibrosis.** (**A**) VEGF mRNA and (**B**) Flk-1 mRNA expression were measured by real-time PCR. Results are expressed as mean ± SD, n = 5 in each group, *P < 0.001 vs SA group; $P < 0.05 vs SA group; #P < 0.05 vs BLM group; +P < 0.001 vs BLM group.

**Figure 5:**
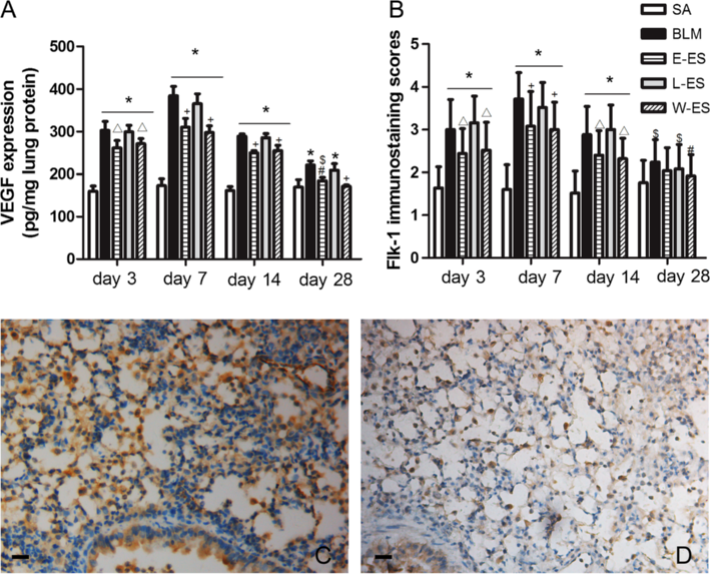
**Effect of endostatin on VEGF/Flk-1 protein expression in BLM-induced pulmonary fibrosis.** (**A**) VEGF concentration in lung tissue lysates and Flk-1 immunostaining scores (**B**) among different experimental groups were presented. Immunohistochemical staining of Flk-1 in lung sections in BLM (**C**) and E-ES (**D**) groups on day 7 (×200) were presented. Results are expressed as mean ± SD, n = 5 in each group, *P < 0.001 vs SA group; $P < 0.05 vs SA group; △P < 0.01 vs BLM group; #P < 0.05 vs BLM group; +P < 0.001 vs BLM group.

VEGF mRNA expression in lung tissue, measured using real-time PCR, increased markedly 3–7 days after BLM instillation. It decreased slightly on day 14 and strongly decreased by day 28 (Figure 4A). The VEGF level in the E-ES and W-ES groups was lower than the BLM group at each stage, as depicted in Figure 4A. The expression of Flk-1 mRNA in rats from the BLM group was higher than in the saline group at each time point after BLM infusion (Figure 4B). Early endostatin administration efficiently inhibited Flk-1 mRNA expression at each timepoint studied compared to the BLM group. However, no differences in the mRNA expression of VEGF or Flk-1 were detected between the L-ES and BLM groups. The changes in the protein levels of VEGF/Flk-1 were similar to the mRNA changes, as assessed by the VEGF concentration in lung tissue lysates (Figure 5A) and Flk-1 immunostaining scores (Figure 5B), which were also reduced in BLM-treated rats by early endostatin treatment at each stage studied.

### Effect of endostatin on TGF-β1 expression in lung tissue lysates

**Figure 6:**
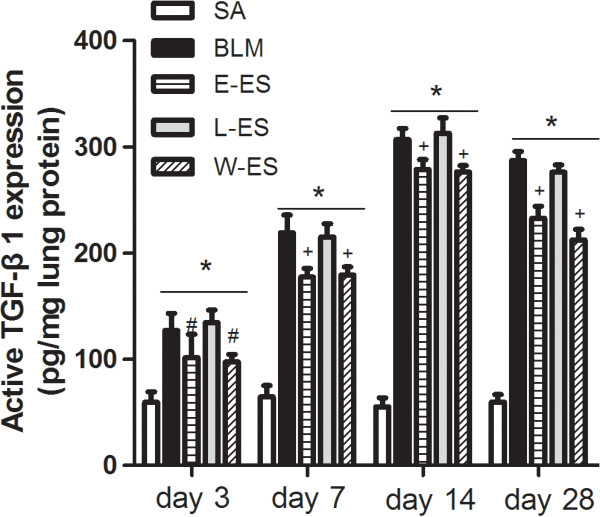
**Effect of endostatin on expresion of TGF-β1 in lung tissue lysates.** TGF-β1 concentrations in lung tissue lysates among different experimental groups were presented. Results are expressed as mean ± SD, n = 5 in each group, *P < 0.001 vs SA group; #P < 0.05 vs BLM group; +P < 0.001 vs BLM group.

TGF-β1 is considered one of the most important fibroblast stimulators leading to pulmonary fibrosis. We measured the TGF-β1 concentration in lung tissue lysates by ELISA. The TGF-β1 concentration increased at all time points examined after BLM challenge. Early endostatin administration reduced BLM-induced TGF-β1 expression on days 3, 7, 14 and 28 after BLM infusion (Figure 6). TGF-β1 Level was lower in the L-ES group than in the BLM group on day 28, although late endostatin treatment did not produce a statistically significant effect (P = 0.142 on day 28).

### Effect of endostatin on ERK1/2 phosphorylation in BLM-induced pulmonary fibrosis

**Figure 7:**
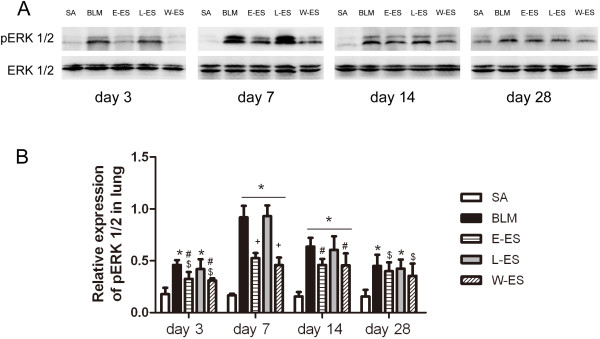
**Endostatin suppressed the ERK1/2 phosphorylation.** (**A**) Western blot showed pERK1/2 protein expression among different experimental groups on days 3, 7, 14 and 28. (**B**) Quantitative densitometry analysis of western blot analysis for pERK1/2. Data are presented as the ratio pERK1/2 and ERK. Results are expressed as mean ± SD, n = 3 in each group, *P < 0.01 vs SA group; $P < 0.05 vs SA group; #P < 0.05 vs BLM group; +P < 0.001 vs BLM group.

ERK activation plays an important role in angiogenesis and inflammation during pulmonary fibrosis. We evaluated whether ERK1/2 phosphorylation levels in rat lungs after BLM challenge were altered by endostatin administration. Western blot analysis showed that BLM challenge caused a significant increase in pERK1/2 compared to saline infusion, whereas endostatin treatment resulted in a significant decrease in pERK1/2 expression (Figure 7). No significant differences were observed in the pERK1/2 expression levels between the L-ES and BLM groups. The total steady-state protein levels remained unchanged.

### Effect of endostatin on alveolar epithelial cell apoptosis

**Figure 8:**
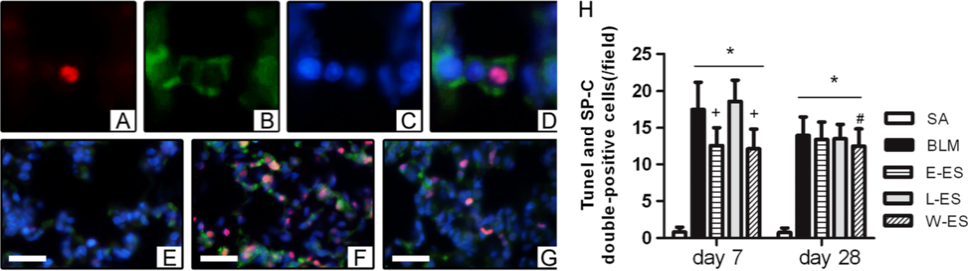
**Effect of endostatin on alveolar epithelial cell apoptosis.** (**A-D**) Representative co-immunofluorescence for TUNEL (**A**), SP-C (**B**) and DAPI (**C**) identifies apoptotic alveolar type II cells (**D, merge**) from a lung section. (**E-G**) Representative fluorescent micrographs for in SA (**E**), BLM (**F**) and E-ES (**G**) groups on day 7 (×400) are shown. (**F**) Quantitative assessment of alveolar type II cell apoptosis among different experimental groups on days 7 and 28 were presented. Bar = 100μm. Results are expressed as mean ± SD, n = 5 in each group, *P < 0.001 vs SA group; #P < 0.05 vs BLM group; +P < 0.001 vs BLM group.

We counted the numbers of SP-C, a marker of alveolar type II cells, and TUNEL double-positive cells in the lung sections to detect apoptosis of alveolar epithelial cells. As shown in Figure 8, BLM induced maximum alveolar type II cell apoptosis on day 7, and the apoptosis decreased on day 28. Early endostatin administration decreased the numbers of apoptotic type II cells on day 7. The whole-course endostatin treatment also produced a similar effect (P = 0.03 on day 28), but there were no significant differences in epithelial cell apoptosis among BLM, E-ES and L-ES groups on day 28.

## Discussion

IPF is an important health problem in humans, and the precise pathogenic mechanisms remain unknown. BLM can cause toxicity by generating an acute inflammatory response and fibrosis in a short period, whereas IPF has a slow and irreversible progression in patients. Histological hallmarks present in BLM-treated rats, such as intra-alveolar buds, mural incorporation of collagen and obliteration of the alveolar space, are similar to IPF patients [36]. The intratracheal instillation of BLM into rodents is widely used as an in vivo experimental model for the study of human pulmonary fibrosis. Emerging evidence has demonstrated that angiogenesis is important in the development and progression of pulmonary fibrosis [8,9,11,13]. In the present study, endostatin was administered at different phases of BLM-induced lung injury and fibrosis in rats. We demonstrated that endostatin treatment had the following effects: (1) decreasing collagen deposition and hydroxyproline content; (2) reducing MVD and VEGF/VEGFR-2 (Flk-1) expression; (3) inhibiting ERK1/2 activation; and (4) reducing the increased number of inflammatory cells present in BALF and decreasing the production of the proinflammatory and fibrotic cytokines TNF-α and TGF-β1; (5) inhibiting the alveolar epithelial cell apoptosis. From these findings, we conclude that endostatin may play an important role in the course of pulmonary fibrosis induced by BLM, which may be mediated through its potentially regulatory effects on inhibiting VEGF receptor and ERK1/2 activation.

Endostatin has potent antiangiogenic and antitumour activity through inhibiting the proliferation and migration of endothelial cells [18]. Endostar, a novel modified human recombinant endostatin with an additional nine amino acids purified from *Escherichia coli*, was approved for the treatment of non–small cell lung cancer in combination with other chemotherapy drugs in China in 2005 [37]. Elevated levels of endostatin are observed in the BALF and serum of IPF patients, especially those with severe respiratory dysfunction, compared to patients without these clinical manifestations [38,39]. A previous study [40] showed that recombinant endostatin inhibited dermal fibrosis in an ex vivo human skin model, and a peptide derived from endostatin prevented dermal and lung fibrosis induced by BLM in a mouse model. The endostatin peptide has also been found to prevent the progression of peritoneal sclerosis, characterised by progressive fibrosis in the peritoneum, in a mouse experimental model [26]. These studies suggest that endostatin has an anti-fibrotic effect and may have therapeutic potential for preventing or reversing organ fibrosis. Similarly, in a study performed by Isobe et al. [27], endostatin expression levels in cardiomyocytes and endostatin serum levels were significantly elevated, and neutralisation of endostatin exacerbated the tissue remodelling and interstitial fibrosis in a rat myocardial infarction model and caused increased tissue collagen and MMP-2/9 activity. Our study found that exogenous endostatin could reduce MVD and collagen deposition in BLM-induced pulmonary fibrosis in rats. On the basis of these reports and our research, we propose that increased endostatin levels in fibrotic lung tissues might establish a negative feedback regulatory loop, which decreases collagen XVIII and MMP-2/9 levels and has a protective effect on the progression of fibrosis. When fibrotic lung tissues are treated with exogenous endostatin, significant positive effects on disease progression should be observed.

Aberrant angiogenesis has been implicated in the development and progression of pulmonary fibrosis. Turner-Warwick [41] originally demonstrated the existence of morphologic neovascularisation in the lungs of IPF patients. Further evidence also strongly suggests a role for angiogenic vascular remodelling in pulmonary fibrosis, and emerging studies indicate that angiogenesis is a central hallmark for the progression of IPF [13,42]. Angiogenesis is regulated by a balance between the angiogenic and angiostatic regulators of blood vessel growth. VEGF is the principal angiogenic factor and is proven to be a proinflammatory and permeability-inducing factor in BLM-induced pulmonary fibrosis [12]. Based on this information, inhibition of the VEGF/VEGFR pathway in PF may have protective effects against angiogenesis and fibrogenesis. Indeed, in an on-going clinical trial, VEGF is the major target for IPF treatment using a tyrosine kinase inhibitor, which is currently in phase 3 (NCT01335464) [43]. Endostatin inhibits VEGF-mediated signalling through a direct interaction with Flk-1 [25,37]. In our study, we used endostatin in the BLM-induced lung fibrosis model. We noted a pronounced decrease in VEGF and Flk-1 expression after endostatin administration, particularly when administered at early stages of disease progression. Meanwhile, the exacerbated MVD was reduced significantly. This finding supports a contribution of VEGF to the fibrotic process via angiogenesis induction [17]. Furthermore, transient protein leakage following BLM can aggravate the initial injury and thereby promote fibrogenesis. VEGF is a major enhancer of vascular permeability [14]. Our study showed that endostatin down-regulated the VEGF-related protein permeability index. In this experiment, we assessed the antiangiogenic efficiency of endostatin and the downregulation of VEGF production in inhibiting BLM-induced pulmonary fibrosis.

ERK is involved in the regulation of lung angiogenesis and inflammation. Recent reports have shown that ERK1/2 activation is increased in an animal model of pulmonary fibrosis, and inhibiting ERK1/2 activation suppresses lung collagen deposition and inflammation and consequently ameliorates pulmonary fibrosis [44,45]. Western blots of human lung biopsy samples also demonstrate significantly increased ERK1/2 signalling in IPF samples compared with normal controls [46]. These findings support ERK1/2 activation playing an important role in pulmonary fibrosis. In our study, we demonstrated that endostatin treatment reduced phosphorylated ERK1/2 overexpression and MVD in the lungs of BLM-treated rats. VEGF overexpression strongly activates ERK1/2, resulting in vascular morphological changes [16]. Endostatin treatment decreases VEGF expression and induces ERK1/2 dephosphorylation, resulting in the retraction of newly formed vessels [47]. Furthermore, TGF-β1 plays a critical role in stimulating myofibroblast differentiation, proliferation and extracellular matrix (ECM) production in pulmonary fibrosis. Our results showed that endostatin reduced TGF-β1 expression in rat lungs after BLM challenge, similar to the results of Sullivan and colleagues, which showed that inhibition of the ERK pathway markedly reduced TGF-β1 expression induced by TNF-α in lung fibroblasts [48].

The inflammatory response was the initial response following BLM administration in our animal model. Angiogenesis is proinflammatory due to enhanced adhesion and permeability in inflammatory lesions [49]. Perivascular inflammatory cell infiltrates are found in the lungs of pulmonary fibrosis patients. Increasing evidence suggests an important role for endostatin in inflammatory reactions. Yin and colleagues [50] found that endostatin gene expression inhibited the development of arthritis, an inflammatory angiogenic disease, in TNF-transgenic mice. Yue L et al. [22] showed that recombinant human endostatin decreased IL-1β and TNF-α levels produced by synoviocytes derived from adjuvant arthritis rats. Consequently, endostatin contributes to the regression of rat adjuvant arthritis, which is related to its anti-angiogenic properties and inhibition of proinflammatory cytokines. Abdollahi and colleagues [51] demonstrated that endostatin inhibits TNF-α-induced angiogenic signalling by downregulating TNFR1 expression. In the present study, endostatin administration not only inhibited aberrant angiogenesis in the lung but also decreased inflammatory cell numbers in the BALF of the BLM model. The anti-inflammatory effect of endostatin may be partly due to the following mechanisms: (1) inhibition of inflammatory cell migration and vascular leakage related to VEGF; (2) attenuation of TNF-α expression, an angiogenic cytokine and one of the most potent proinflammatory cytokines promoting inflammatory cell infiltration in the rat lung; and (3) decreased TGF-β1 levels and inhibition of inflammatory cell chemotaxis in the lung. These results suggest that endostatin treatment may alter the course of the inflammatory process associated with pulmonary fibrosis.

Given the important role of epithelial cell apoptosis in human IPF [52], alveolar type II cells apoptosis was detected in our study. Richter et al. [39] indicated that endostatin reduces epithelial proliferation and wound repair in a FasL and caspase dependent manner in vitro. However, we here showed that BLM-induced pulmonary injury and alveolar type II apoptosis were suppressed in endostatin treated rats in the early stage. Inflammation can lead to increased alveolar epithelial cell apoptosis [53]. The epithelium-protective effect of endostatin in the early stage may be secondary to the reduced vascular permeability and decreased inflammation in vivo experiments. Furthermore, maybe it related to the reduced level of TGF-β1, which is an enhancer of Fas-mediated apoptosis of lung epithelial cells [54]. Additionally, although it was reported that physiological doses of endostatin reduced epithelial cell wound repair in vitro [39], in our in vivo experiment, exogenous endostatin used in microgram amounts, a supraphysiological doses, produced a protective effect on epithelial cell apoptosis in rat model of pulmonary fibrosis. The effects of endostatin on these mechanisms warrant further investigation.

## Conclusion

Taken together, endostatin plays a complex role in BLM-induced pulmonary fibrosis. Our findings demonstrated that endostatin blocked VEGF/Flk-1 signalling and diminished ERK1/2 phosphorylation, inhibiting the increased MVD. Meanwhile, endostatin decreased TNF-α and TGF-β1 production, leukocyte trafficking and protein leakage. Furthermore, endostatin decreased alveolar epithelial cell apoptosis. Our results support the hypothesis that early endostatin administration attenuates the progression of pulmonary fibrosis by inhibiting angiogenesis, inflammation and epithelial cell apoptosis. The pathogenesis of pulmonary fibrosis is likely complex, involving multiple pathways. Endostatin treatment ameliorated BLM-induced pulmonary fibrosis but was not able to completely prevent or reverse the fibrotic process. Further studies in existing fibrosis models are necessary.

## Abbreviations

IPF: Idiopathic pulmonary fibrosis; BLM: Bleomycin; ERK1/2: Extracellular signal-regulated protein kinases 1 and 2; VEGF: Vascular endothelial growth factor; VEGFR2 (Flk-1): Vascular endothelial growth factor receptor 2; TNF-α: Tumor necrosis factor-α; MMP: Metalloproteinase; BAL: Bronchoalveolar lavage; PPI: Protein permeability index; MVD: Microvascular density; ELISA: Enzyme-linked immunosorbent assay; TGF-β1: Transforming growth factor β1; ECM: Extracellular matrix; SA: Saline.

## Competing interests

The authors declare that they have no competing interests.

## Authors’ contributions

WYY and LDJ designed the study. WYY performed the statistical analyses and wrote the manuscript. GHS, KYM, WYY, YZH and LXL carried out the animal experiments. TGY and LQH participated in the study design and coordination and helped to correct the manuscript. All authors read and approved the final manuscript.

## References

[B1] Gross TJ Hunninghake GW Idiopathic pulmonary fibrosis N Engl J Med 2001 345 517 525 10.1056/NEJMra003200 11519507

[B2] American Thoracic Society Idiopathic pulmonary fibrosis: diagnosis and treatment. International consensus statement. American Thoracic Society (ATS), and the European Respiratory Society (ERS) Am J Respir Crit Care Med 2000 161 646 664 10673212 10.1164/ajrccm.161.2.ats3-00

[B3] Panos RJ Mortenson RL Niccoli SA King TE Jr Clinical deterioration in patients with idiopathic pulmonary fibrosis: causes and assessment Am J Med 1990 88 396 404 10.1016/0002-9343(90)90495-Y 2183601

[B4] Mason RJ Schwarz MI Hunninghake GW Musson RA NHLBI Workshop Summary. Pharmacological therapy for idiopathic pulmonary fibrosis. Past, present, and future Am J Respir Crit Care Med 1999 160 1771 1777 10.1164/ajrccm.160.5.9903009 10556155

[B5] Ota K Diagnosis and treatment of idiopathic pulmonary fibrosis Nihon Naika Gakkai Zasshi 2007 96 557 561 10.2169/naika.96.557 17419427

[B6] King TE Jr Pardo A Selman M Idiopathic pulmonary fibrosis Lancet 2011 378 1949 1961 10.1016/S0140-6736(11)60052-4 21719092

[B7] Peao MN Aguas AP de Sa CM Grande NR Neoformation of blood vessels in association with rat lung fibrosis induced by bleomycin Anat Rec 1994 238 57 67 10.1002/ar.1092380108 7509580

[B8] Keane MP Belperio JA Arenberg DA Burdick MD Xu ZJ Xue YY Strieter RM IFN-gamma-inducible protein-10 attenuates bleomycin-induced pulmonary fibrosis via inhibition of angiogenesis J Immunol 1999 163 5686 5692 10553099

[B9] Burdick MD Murray LA Keane MP Xue YY Zisman DA Belperio JA Strieter RM CXCL11 attenuates bleomycin-induced pulmonary fibrosis via inhibition of vascular remodeling Am J Respir Crit Care Med 2005 171 261 268 10.1164/rccm.200409-1164OC 15502109

[B10] Hamada N Kuwano K Yamada M Hagimoto N Hiasa K Egashira K Nakashima N Maeyama T Yoshimi M Nakanishi Y Anti-vascular endothelial growth factor gene therapy attenuates lung injury and fibrosis in mice J Immunol 2005 175 1224 1231 16002726 10.4049/jimmunol.175.2.1224

[B11] Ou XM Li WC Liu DS Li YP Wen FQ Feng YL Zhang SF Huang XY Wang T Wang K VEGFR-2 antagonist SU5416 attenuates bleomycin-induced pulmonary fibrosis in mice Int Immunopharmacol 2009 9 70 79 10.1016/j.intimp.2008.10.002 18976720

[B12] Tabata C Tabata R Kadokawa Y Hisamori S Takahashi M Mishima M Nakano T Kubo H Thalidomide prevents bleomycin-induced pulmonary fibrosis in mice J Immunol 2007 179 708 714 17579094 10.4049/jimmunol.179.1.708

[B13] Wang X Zhu H Yang X Bi Y Cui S Vasohibin attenuates bleomycin induced pulmonary fibrosis via inhibition of angiogenesis in mice Pathology 2010 42 457 462 10.3109/00313025.2010.493864 20632823

[B14] Ferrara N Gerber HP LeCouter J The biology of VEGF and its receptors Nat Med 2003 9 669 676 10.1038/nm0603-669 12778165

[B15] Ziche M Morbidelli L Choudhuri R Zhang HT Donnini S Granger HJ Bicknell R Nitric oxide synthase lies downstream from vascular endothelial growth factor-induced but not basic fibroblast growth factor-induced angiogenesis J Clin Invest 1997 99 2625 2634 10.1172/JCI119451 9169492 PMC508108

[B16] Parenti A Morbidelli L Cui XL Douglas JG Hood JD Granger HJ Ledda F Ziche M Nitric oxide is an upstream signal of vascular endothelial growth factor-induced extracellular signal-regulated kinase1/2 activation in postcapillary endothelium J Biol Chem 1998 273 4220 4226 10.1074/jbc.273.7.4220 9461619

[B17] Farkas L Farkas D Ask K Moller A Gauldie J Margetts P Inman M Kolb M VEGF ameliorates pulmonary hypertension through inhibition of endothelial apoptosis in experimental lung fibrosis in rats J Clin Invest 2009 119 1298 1311 10.1172/JCI36136 19381013 PMC2673845

[B18] O'Reilly MS Boehm T Shing Y Fukai N Vasios G Lane WS Flynn E Birkhead JR Olsen BR Folkman J Endostatin: an endogenous inhibitor of angiogenesis and tumor growth Cell 1997 88 277 285 10.1016/S0092-8674(00)81848-6 9008168

[B19] Wickstrom SA Veikkola T Rehn M Pihlajaniemi T Alitalo K Keski-Oja J Endostatin-induced modulation of plasminogen activation with concomitant loss of focal adhesions and actin stress fibers in cultured human endothelial cells Cancer Res 2001 61 6511 6516 11522648

[B20] Dhanabal M Ramchandran R Waterman MJ Lu H Knebelmann B Segal M Sukhatme VP Endostatin induces endothelial cell apoptosis J Biol Chem 1999 274 11721 11726 10.1074/jbc.274.17.11721 10206987

[B21] Becker CM Sampson DA Rupnick MA Rohan RM Efstathiou JA Short SM Taylor GA Folkman J D'Amato RJ Endostatin inhibits the growth of endometriotic lesions but does not affect fertility Fertil Steril 2005 84 Suppl 2 1144 1155 16210006 10.1016/j.fertnstert.2005.04.040

[B22] Yue L Wang H Liu LH Shen YX Wei W Anti-adjuvant arthritis of recombinant human endostatin in rats via inhibition of angiogenesis and proinflammatory factors Acta Pharmacol Sin 2004 25 1182 1185 15339395

[B23] Tolstanova G Deng X Khomenko T Garg P Paunovic B Chen L Sitaraman SV Shiloach J Szabo S Sandor Z Role of anti-angiogenic factor endostatin in the pathogenesis of experimental ulcerative colitis Life Sci 2011 88 74 81 10.1016/j.lfs.2010.10.026 21047522 PMC9552542

[B24] Hajitou A Grignet C Devy L Berndt S Blacher S Deroanne CF Bajou K Fong T Chiang Y Foidart JM Noel A The antitumoral effect of endostatin and angiostatin is associated with a down-regulation of vascular endothelial growth factor expression in tumor cells FASEB J 2002 16 1802 1804 12354694 10.1096/fj.02-0109fje

[B25] Kim YM Hwang S Pyun BJ Kim TY Lee ST Gho YS Kwon YG Endostatin blocks vascular endothelial growth factor-mediated signaling via direct interaction with KDR/Flk-1 J Biol Chem 2002 277 27872 27879 10.1074/jbc.M202771200 12029087

[B26] Tanabe K Maeshima Y Ichinose K Kitayama H Takazawa Y Hirokoshi K Kinomura M Sugiyama H Makino H Endostatin peptide, an inhibitor of angiogenesis, prevents the progression of peritoneal sclerosis in a mouse experimental model Kidney Int 2007 71 227 238 10.1038/sj.ki.5002040 17191085

[B27] Isobe K Kuba K Maejima Y Suzuki J Kubota S Isobe M Inhibition of endostatin/collagen XVIII deteriorates left ventricular remodeling and heart failure in rat myocardial infarction model Circ J 2010 74 109 119 10.1253/circj.CJ-09-0486 19966499

[B28] Ashcroft T Simpson JM Timbrell V Simple method of estimating severity of pulmonary fibrosis on a numerical scale J Clin Pathol 1988 41 467 470 10.1136/jcp.41.4.467 3366935 PMC1141479

[B29] Otsuka M Takahashi H Shiratori M Chiba H Abe S Reduction of bleomycin induced lung fibrosis by candesartan cilexetil, an angiotensin II type 1 receptor antagonist Thorax 2004 59 31 38 10.1136/thx.2003.000893 14694243 PMC1758867

[B30] Weidner N Intratumor microvessel density as a prognostic factor in cancer Am J Pathol 1995 147 9 19 7541613 PMC1869874

[B31] Shimizu M Saitoh Y Itoh H Immunohistochemical staining of Ha-ras oncogene product in normal, benign, and malignant human pancreatic tissues Hum Pathol 1990 21 607 612 10.1016/S0046-8177(96)90006-4 2161789

[B32] Perkins GD Chatterjie S McAuley DF Gao F Thickett DR Role of nonbronchoscopic lavage for investigating alveolar inflammation and permeability in acute respiratory distress syndrome Crit Care Med 2006 34 57 64 10.1097/01.CCM.0000190197.69945.C5 16374157

[B33] Livak KJ Schmittgen TD Analysis of relative gene expression data using real-time quantitative PCR and the 2(−Delta Delta C(T)) Method Methods 2001 25 402 408 10.1006/meth.2001.1262 11846609

[B34] Lee SH Jang AS Kim YE Cha JY Kim TH Jung S Park SK Lee YK Won JH Kim YH Park CS Modulation of cytokine and nitric oxide by mesenchymal stem cell transfer in lung injury/fibrosis Respir Res 2010 11 16 10.1186/1465-9921-11-16 20137099 PMC2827393

[B35] Imazu Y Yanagi S Miyoshi K Tsubouchi H Yamashita S Matsumoto N Ashitani J Kangawa K Nakazato M Ghrelin ameliorates bleomycin-induced acute lung injury by protecting alveolar epithelial cells and suppressing lung inflammation Eur J Pharmacol 2011 672 153 158 10.1016/j.ejphar.2011.09.183 21996315

[B36] Usuki J Fukuda Y Evolution of three patterns of intra-alveolar fibrosis produced by bleomycin in rats Pathol Int 1995 45 552 564 10.1111/j.1440-1827.1995.tb03503.x 7496500

[B37] Ling Y Yang Y Lu N You QD Wang S Gao Y Chen Y Guo QL Endostar, a novel recombinant human endostatin, exerts antiangiogenic effect via blocking VEGF-induced tyrosine phosphorylation of KDR/Flk-1 of endothelial cells Biochem Biophys Res Commun 2007 361 79 84 10.1016/j.bbrc.2007.06.155 17644065

[B38] Sumi M Satoh H Kagohashi K Ishikawa H Sekizawa K Increased serum levels of endostatin in patients with idiopathic pulmonary fibrosis J Clin Lab Anal 2005 19 146 149 10.1002/jcla.20069 16025479 PMC6807939

[B39] Richter AG McKeown S Rathinam S Harper L Rajesh P McAuley DF Heljasvaara R Thickett DR Soluble endostatin is a novel inhibitor of epithelial repair in idiopathic pulmonary fibrosis Thorax 2009 64 156 161 10.1136/thx.2008.102814 18852160

[B40] Yamaguchi Y Takihara T Chambers RA Veraldi KL Larregina AT Feghali-Bostwick CA A peptide derived from endostatin ameliorates organ fibrosis Sci Transl Med 2012 4 136ra171 10.1126/scitranslmed.3003421 PMC5064443 22649092

[B41] Turner-Warwick M Precapillary Systemic-Pulmonary Anastomoses Thorax 1963 18 225 237 10.1136/thx.18.3.225 14064617 PMC1018768

[B42] Farkas L Gauldie J Voelkel NF Kolb M Pulmonary hypertension and idiopathic pulmonary fibrosis: a tale of angiogenesis, apoptosis, and growth factors Am J Respir Cell Mol Biol 2011 45 1 15 10.1165/rcmb.2010-0365TR 21057104

[B43] Richeldi L Costabel U Selman M Kim DS Hansell DM Nicholson AG Brown KK Flaherty KR Noble PW Raghu G Efficacy of a tyrosine kinase inhibitor in idiopathic pulmonary fibrosis N Engl J Med 2011 365 1079 1087 10.1056/NEJMoa1103690 21992121

[B44] Galuppo M Esposito E Mazzon E Di Paola R Paterniti I Impellizzeri D Cuzzocrea S MEK inhibition suppresses the development of lung fibrosis in the bleomycin model Naunyn Schmiedebergs Arch Pharmacol 2011 384 21 37 10.1007/s00210-011-0637-7 21533992

[B45] Madala SK Schmidt S Davidson C Ikegami M Wert S Hardie WD MEK-ERK pathway modulation ameliorates pulmonary fibrosis associated with epidermal growth factor receptor activation Am J Respir Cell Mol Biol 2012 46 380 388 10.1165/rcmb.2011-0237OC 22021337 PMC3326433

[B46] Yoshida K Kuwano K Hagimoto N Watanabe K Matsuba T Fujita M Inoshima I Hara N MAP kinase activation and apoptosis in lung tissues from patients with idiopathic pulmonary fibrosis J Pathol 2002 198 388 396 10.1002/path.1208 12375272

[B47] Schmidt A Wenzel D Thorey I Sasaki T Hescheler J Timpl R Addicks K Werner S Fleischmann BK Bloch W Endostatin influences endothelial morphology via the activated ERK1/2-kinase endothelial morphology and signal transduction Microvasc Res 2006 71 152 162 10.1016/j.mvr.2006.01.001 16650878

[B48] Sullivan DE Ferris M Pociask D Brody AR Tumor necrosis factor-alpha induces transforming growth factor-beta1 expression in lung fibroblasts through the extracellular signal-regulated kinase pathway Am J Respir Cell Mol Biol 2005 32 342 349 10.1165/rcmb.2004-0288OC 15653932

[B49] Moulton KS Melder RJ Dharnidharka VR Hardin-Young J Jain RK Briscoe DM Angiogenesis in the huPBL-SCID model of human transplant rejection Transplantation 1999 67 1626 1631 10.1097/00007890-199906270-00020 10401773

[B50] Yin G Liu W An P Li P Ding I Planelles V Schwarz EM Min W Endostatin gene transfer inhibits joint angiogenesis and pannus formation in inflammatory arthritis Mol Ther 2002 5 547 554 10.1006/mthe.2002.0590 11991745

[B51] Abdollahi A Hahnfeldt P Maercker C Grone HJ Debus J Ansorge W Folkman J Hlatky L Huber PE Endostatin's antiangiogenic signaling network Mol Cell 2004 13 649 663 10.1016/S1097-2765(04)00102-9 15023336

[B52] Thannickal VJ Horowitz JC Evolving concepts of apoptosis in idiopathic pulmonary fibrosis Proc Am Thorac Soc 2006 3 350 356 10.1513/pats.200601-001TK 16738200 PMC2231523

[B53] Drakopanagiotakis F Xifteri A Polychronopoulos V Bouros D Apoptosis in lung injury and fibrosis Eur Respir J 2008 32 1631 1638 10.1183/09031936.00176807 19043009

[B54] Hagimoto N Kuwano K Inoshima I Yoshimi M Nakamura N Fujita M Maeyama T Hara N TGF-beta 1 as an enhancer of Fas-mediated apoptosis of lung epithelial cells J Immunol 2002 168 6470 6478 12055267 10.4049/jimmunol.168.12.6470

